# Supplementary material for: Are carbon emissions trading and green financial instruments synergistic? -Comprehensive quantitative research based on content analysis
Source: PLoS One. 2024 Mar 7;19(3):e0298601. doi: 10.1371/journal.pone.0298601 (PMC10919601; doi:10.1371/journal.pone.0298601)
Supplement: S1 Data — (ZIP) [file pone.0298601.s001.zip › Data/地级市Z_F工作报告.zip!/╣π╢1⁄2/├»├√╩╨/└┤╫╘╣1⁄2╓┌║┼í╛┬φ┐╦╩2╛▌═°í┐_2015.docx]

各位代表：

我代表市人民政府向大会作工作报告，请予审议，并请政协委员和其他列席人员提出意见。

一、2014年工作回顾

2014年，茂名走过了不平凡的一年，大事多、难事多。在省委、省政府和市委的坚强领导下，在市人大及其常委会和市政协的监督支持下，市政府全面贯彻党的十八大、十八届三中、四中全会精神，认真落实粤东西北地区振兴发展战略部署，全力以赴抓项目、促落实，千方百计稳增长、惠民生。面对经济下行压力增大和重大事件冲击等困难和挑战，始终保持定力、主动作为，实现了经济社会持续稳步发展，在全省粤东西北地区振兴发展评估考核中，取得总分第一的优异成绩。全市完成生产总值2349亿元，增长10.4%。公共财政预算收入100.4亿元，增长11.1% ；城镇居民人均可支配收入22040元，增长10%，农民人均纯收入11881元，增长11%。

一年来，我们做的主要工作及成效如下：

（一）全力以赴保增长，经济运行稳中向好。

综合竞争力进一步提升。主要经济指标争先进位，20个重要经济指标增速中，有9个位居全省前4，其中生产总值增速位居第4、规上工业增加值增速位居第3、固定资产投资增速位居第4、实际利用外资增速位居第1、出口总额增速位居第3、全社会用电量增速位居第3。

投资实现快速增长。完成固定资产投资850.6亿元，增长 36.1%，其中工业投资增长30.3%，交通建设投资增长79.4%。省、市重点项目分别完成投资计划118%和95.1%，同比提高5.4和7.7个百分点。信贷投放力度加大，年末人民币贷款余额748.9亿元，增长17.6%；存贷比为42.4%，提高1.7个百分点。

市场消费保持稳定。完成社会消费品零售总额1113.9亿元，增长10.4%。加快引进和建设城市综合体。举办系列展销活动，促进汽车、房产等商品消费。推动茂名水果网上销售，全市电商平台荔枝、龙眼销售量同比增长5倍。开展李花节、荔枝节等特色活动，全年接待游客1487.1万人，同比增长28.9%。

工业生产较快发展。全市实现规上工业增加值712.2亿元，增长16.8%，其中地方规上工业增加值374.5亿元，增长18.2%。支持茂名石化公司发展，该公司加工原油1803万吨，增长10.6%；乙烯产量114万吨，居全国之首。推进园区扩能增效，3个省级园区实现规上工业增加值77.1亿元，同比增长20.9%。

农业产业化进程加快。新增国家级农民专业合作社示范社12家，合作社总量全省第一。省水果产业带项目落户茂名，全国500强企业海亮集团千亩种植基地落户高州；粤西农批首期实现对外招商；新建一批农产品冷链配送物流项目。新建钢质渔船80艘，成为我省拥有钢体渔船最多的市。

（二）突出开展交通大会战，基础设施建设取得重大突破。

对外交通建设强力推进。包茂高速茂名段进入路面施工阶段。汕湛高速茂名先行段开工建设。云浮至茂名高速公路纳入省高速公路网规划。深茂铁路江门至茂名段全线开工，征地拆迁工作进展较快。湛江机场迁建已启动项目可研报告编制工作。

对内交通条件加快改善。13个重点交通项目开展冬季劳动竞赛，创多年来茂名交通建设项目开工、在建数量之最。茂化快线路面改造段已完成，改线段完成工程量过半。G207信宜至高州段路面改造工程、G325茂名段改造工程开工建设。海洋大道东段加快推进。茂名港大道基本建成通车。开通沈海高速鳌头出口。山根公路改造项目路面主体工程春节前将基本完工。完成县道改造89.5公里，农村公路路面硬底化611公里。45座地养危桥改造完成37座，在建8座。

港口建设稳步展开。博贺新港区东防波堤推进1065米，西防波堤开工建设。博贺新港区30万吨级航道（首期15万吨级）工程编制完成预可研报告。粤电博贺煤炭码头项目护岸及防波堤工程完成1700米。博贺新港区通用码头工程岸线使用获批。

环境治理能力进一步提升。河西城区生活污水处理厂完成厂区土建工程和设备安装，高州水库库区7个镇污水处理站全部建成投用，高州金山污水处理厂进入调试阶段。依法关闭高州水库集雨区302家养殖场。茂名石化炼油厂和茂名热电厂一批治理减排工程建成投用。打击盗挖露天焚烧油页岩矿渣和违法排污行为，严厉查处公馆“1·10”环境污染事件，全年移送涉嫌环境犯罪案件4宗。

水利电力基础设施建设扎实推进。民生水利工程建设完成投资4.8亿元。完成大型灌区改造渠道153公里，改善灌溉面积63.3万亩。电网工程建设完成投资5.9亿元，完成900个“应改未改”农网改造项目。

（三）做实发展平台，开创滨海发展新格局。

进一步强化“三大平台”功能定位，加快基础设施建设，滨海新区走在粤东西北新区发展前列。

滨海新区起步区全面开发建设。博贺湾大道建设进展顺利。恒大路改建工程开工。中海油LNG项目获批开展前期工作。粤电博贺发电项目加快推进。保利东海岸项目和龙头湾旅游综合开发项目分别注册成立项目公司。全年完成投资36.5亿元，增长105.8%；实现融资10.9亿元，新引入项目10个。

高新区实现跨越发展。德国巴斯夫异壬醇项目进入全面建设阶段，聚丙烯项目投料试产、华南塑化五金机电汽配商贸城项目抓紧推进。西南片区4平方公里基础设施建设基本成型，工业大道一期建成通车，高新大道、公用管廊加快推进，17个项目进场施工。全年开发建设投入12.8亿元，新开工项目25个。

水东湾新城建设稳步推进。全面启动水东湾综合整治开发项目，水东湾大桥钢栈桥进入施工阶段，水东湾清淤工程全面开工。高地智慧城建设加快推进，茂名职业技术学院新校区一期主体工程完工，市人民医院水东湾新城院区项目完成征地256亩。引入广州科技职业技术学院粤西校区项目。

（四）推动新型城镇化，城乡面貌有效改善。

茂港、电白成功合并设立电白区，中心城区规模大幅扩大。加强城区公共基础设施建设，城乡面貌加快改善。

中心城区路网等基础设施进一步完善。官渡三路路基春节前将拉通。完成油城一二三路改造工程，文冲口铁路桥底水浸问题有效解决；完成天桥路和站南路改造工程，人民南五六路动工建设；油城十路建成通车，茂东快线动工建设，提升了城市西、南和东三个出口城市形象。实施站前三四路、官渡一二路等15个路段沥青罩面工程。高山桥改造工程启动，矿业北路完成改造。电白城区人民路、厂前路、新湖路改造工程动工。

城市生活质量全面提升。完成海洋大道（一期）和榕园等绿化工程，新增绿地面积3.3万平方米，建成区绿化覆盖率达42.7%。投资1.4亿元新建河东水厂第二条供水管线和改造出水管线，有效解决了市区供水不足问题。开通城市北组团至南组团和东组团公交线路，推动三大组团同城化发展。按照“引水、种树、建馆、修路”思路，打造具有茂名特色的露天矿生态公园，引水工程和道路建设项目开工，完成矿区2000亩植被恢复任务。河西老工业区纳入全国老工业区搬迁改造试点。建成大塘农贸市场，疏导解决了周边占道经营问题。电白城区东、西湖改造取得明显成效。出台政策推动既有住宅加装电梯。

城乡环境持续改善。划定茂名市高污染燃料禁燃区，淘汰黄标车、老旧汽车19769辆；推进市区大气PM10、PM2.5实时监测，市区空气质量指数排名位居全省前列。完成造林作业面积17.3万亩，加密加宽高速公路景观林带50公里，绿化国道300多公里，绿化补植茂名大道、茂名港大道。云开山被确立为国家级自然保护区。深入推进城乡清洁工程，全年投入资金1.4亿元，初步建立城乡生活垃圾收运处理体系，环境卫生明显改善。

（五）深化改革创新，发展动力和活力不断增强。

行政体制改革进一步深化。启动编制部门纵向权责清单试点工作。经济功能区体制进一步理顺。简政强镇事权改革优化完善。市县两级政府机构改革基本完成。统筹推进商事制度改革，新登记企业户数和注册资本分别增长9.5%和36.9%。全市综合政务服务进一步完善，公共资源交易范围进一步拓展。

财政体制改革深入推进。进一步完善市与电白区财政体制、市级重点建设项目税费分成办法等，市级转移支付和补助区（市）资金11.8亿元。市级试行零基预算编审改革。大额专项资金一半以上安排项目建设。清理盘活财政存量资金。推进部门预决算和“三公”经费等信息公开，“三公”经费下降23.9%。

投融资和金融改革迈出新步伐。市管权限投资项目实行备案制。拓展融资渠道，市交投集团发行10亿元企业债，茂名港集团发行5亿元中期票据，高新区3.8亿元城镇化贷款到位。成功申报省股权基金9.3亿元。注重防范债务风险，出台市级政府性债务管理暂行办法。设立粤西首家村镇银行。

创新项目建设和推进的工作方式方法。借鉴包茂高速茂名段建设经验做法，建立“三倒逼”项目管理机制和“六个一”工作督导机制；推广“大包干”机制，重心下移推进征地拆迁；倡导“双标”管理，加强项目质量管理。建立完善“两会两考两通报”制度，推动全市形成“比学赶帮超”浓厚氛围。

（六）坚持以人为本，民生社会事业加快发展。

民生实事取得新进展。全市拨付省十件民生实事市县级配套资金30.4亿元，支出进度居全省第一。新开工建设保障性住房2075套，基本建成2244套。积极发展养老服务事业，全年新增养老床位7030张。扶贫开发进展顺利，投入帮扶资金11.5亿元，有劳动力贫困户家庭人均纯收入全部超5220元。低收入住房困难户、“两不具备”村庄搬迁户和因灾全倒户住房建设（改造）竣工率均达100%。完成58个地质灾害隐患点搬迁治理。

就业和社会保障工作进一步加强。就业形势稳定，城镇新增就业71286人，城镇失业人员再就业17740人。企业职工养老保险参保率达87%。企业退休人员基本养老金月人均增加286.2元。城乡居民养老保险基础养老金提至80元；城乡居民医保筹资标准提至370元。启动城乡居民大病保险和城镇职工补充医疗保险。城乡低保月人均补差分别提高93元、41元，农村五保集中和分散供养年人均分别提高347元、725元，孤儿集中和分散供养生活保障标准月人均分别提高150元、100元。

科教文卫发展水平不断提高。成功申报国家知识产权试点城市，获省科学技术奖项数居粤东西北地区第一，专利授权增长率居全省第二，成立了一批新型研发机构。加快教育创强，累计投入创强资金45亿元，77个镇（街）通过省督导验收或督前检查。全市山区、农村边远地区义务教育学校教师岗位津贴全面落实。高考上本科线人数再创新高，占全省上线人数十分之一。第二高级技工学校升格为茂名技师学院。广东石油化工学院扩建征地累计落实415亩。登革热等疫情防控工作扎实有力。完成县级公立医院改革试点工作，全市新增民营医疗机构19间。“茂名大讲堂”、“悦动茂名”等文化品牌深受群众欢迎。市图书馆成功创建国家一级图书馆。整治和改造市体育中心，成功举办市第十二届运动会。

平安茂名建设持续深化。集中开展“六大专项”打击整治行动，有效整治治安重点地区和治安突出问题；推广“以民促安”打防体系经验，推进市区安保点建设和“平安家居”社会视频安装整合。深入开展“一村（居）一律师”工作。开展油气管道、危化品运输、道路交通等行业领域专项整治，全市安全生产形势总体平稳。此外，人口计生、食品药品、民族宗教、统计、物价、国防动员、人防、双拥、气象、地震、残联、红十字、外事、侨务、档案、方志等工作取得新的成效。

同时，政府自身建设不断加强。自觉接受市人大及其常委会和市政协监督，办理人大代表建议84件、政协委员提案310件。政务公开深入推进，网络问政实现常态化发展。审计服务发展取得新突破。党风廉政建设责任制全面落实，纠风治乱和案件查处力度加大。庸懒散奢等不良风气得到有效整治，机关行政效能明显提升。全年问责纪律松驰、作风不正及工作不力的干部97人。深入开展党的群众路线教育实践活动，大力整治“四风”突出问题，一批与民攸关的热点难点问题得到解决。

去年政府工作报告确定的118项工作，有110项按期完成，有8项正在稳步推进。对没能按时完成的事项，我们将切实加大推进力度，逐项跟踪落实。

各位代表！过去一年，我市取得的成绩来之不易。这是在省委、省政府和市委的正确领导下，全市人民齐心协力、奋力拼搏的结果。在此，我代表市政府向全市人民，向驻茂人民解放军、武警官兵和各民主党派、人民团体、社会各界人士致以崇高敬意！向所有关心支持茂名发展的港澳同胞、台湾同胞、海外侨胞及国际友人，表示衷心的感谢！

我们也清醒认识到，年初确定的部分经济增长目标未能如期实现；工业持续发展后劲不足，产业新增长点不多；项目建设瓶颈因素依然存在，重大项目支撑乏力；中心城区集聚力、辐射力不强，城市经营和管理水平不高；污染偷排现象时有发生，环境质量有待改善；可支配财力不足；民生保障水平与人民群众的期待仍有较大差距，社会管理和公共服务体系有待完善；政府公信力有待进一步提升，干事创业精神需要进一步提振等。对此，我们将高度重视，认真解决。

二、2015年工作安排

各位代表！2015年是全面深化改革的关键之年，是全面落实依法治国方略的开局之年，也是全面完成“十二五”规划的收官之年，做好今年工作意义重大。当前，世界经济仍处在国际金融危机后的深度调整期，总体复苏疲弱态势难有明显改观。我国经济发展进入中高速运行的新常态，国内“三期叠加”阶段仍然面临不少不稳定不确定因素，经济下行压力和潜在风险并存。具体到我市，我们正处于爬坡过坎的关键时期。包茂高速、深茂铁路、汕湛高速等重大基础设施相继动工，各区（市）及“三大平台”扩容提质和扩能增效建设全面推进，全市步入投资高峰期，而短期内项目的产出效益较少，资金压力较大，各方面面临的困难很多。但同时也要看到，只要我们咬紧牙关，再苦干实干3-5年，利用好当前国际大宗商品价格走低和省加大交通投资力度的机遇，完善全市立体交通网络；利用好当前产业转移和扩能增效的机遇，努力培育发展新优势、做大做强现代产业；利用好国家、省推进新型城镇化的机遇，拉开城市框架、做旺滨海新城人气，茂名将迎来新的发展格局和新的发展热潮。

2015年政府工作的总体要求是：全面贯彻落实党的十八届四中全会、省委十一届四次全会和省进一步促进粤东西北地区振兴发展工作会议精神，按照市委十届六次全会的部署要求，围绕人均GDP赶超全国平均水平的目标，坚持加快发展主旋律和稳中求进总基调，构建“三大平台”和县域发展两大主战场，持续用劲抓好“三大抓手”，推进改革创新，实现绿色发展，办好民生实事，完成好“十二五”规划目标，谋划好“十三五”规划工作，推动茂名振兴发展。

今年经济社会发展主要预期目标是：生产总值增长10%左右，固定资产投资增长20%，社会消费品零售总额增长10%，公共财政预算收入增长10.5%，居民消费价格涨幅控制在3%左右；居民收入增长与经济增长基本同步，进出口总额和节能减排约束性指标完成省下达年度计划。经过全市上下的共同努力，力争全年生产总值突破2500亿元，固定资产投资总额突破1000亿元，出口总额突破10亿美元，全社会用电量突破100亿千瓦时，高速公路通车里程突破200公里。突出抓好8个方面工作：

（一）稳中求进推动经济发展。

大力培育新增长点。全力支持茂名石化公司新上项目，启动茂名石化液体化工码头及配套储运设施建设。积极参股建设茂名石化高润南方公司，力促南海精细化工有限公司增加地方税收。大力发展新兴产业，谋划建设太阳能发电项目。加快推进市民片区粤西农批、亿丰家居等物流重点项目建设，打造粤西物流集聚园区。围绕产业、临港、矿产资源、劳动力和乡贤等优势，加大招商引资力度。抢抓今年底包茂高速全线开通机遇，启动农业、旅游、房地产等产业招商工作。注重招商选资和项目服务，提高单位土地投资强度和产出效益。鼓励投资额大、带动性强的技术改造项目申报省级、国家专项资金。

全力推进重点项目建设。加强项目储备，保障重点项目前期工作经费。实施“一事一策一团队”工作机制，强化市领导挂钩督办制度，定期召开重点项目联席审批会议。总结推广包茂高速茂名段项目管理经验，借鉴推广市交通运输局和滨海新区将项目移交国有企业管理的做法。进一步加强政府投资项目招投标管理和监督，吸引更多有实力、信誉好的勘察设计、施工和监理队伍。

深挖消费潜力。发展壮大限额以上企业，支持商贸企业通过重组兼并等方式做大做强。合理布局一批特色美食街区、步行街及城市综合体。进一步整合利用国有资产，大力支持电子商务、自主创业等。发挥供销系统渠道作用，积极扩大和利用农村销售网点。支持4G基站建设，加快光纤入户宽带网络基础设施建设，促进信息消费。大力谋划各类促销展会。积极拓展健康、养老、文化等服务业发展。加快成立市电子商务协会，组织电商人才培训，推动“茂货网上行”。推进旅游项目建设,统筹整合旅游线路,积极引客入茂。

（二）依托平台大力发展实体经济。

加快滨海新区起步区开发建设。全面推进吉达港区工业园区、博贺临港工业园区基础设施建设。加快粤电博贺电厂核准和建设，力争年内建成投产。配合推动中海油LNG项目前期工作，全面启动项目围海造地工程。加快建设中化液体化工仓储项目、博贺国际旅游岛、东达龙头湾项目。

继续做大做强高新区。围绕创建国家级高新区目标，加快开发建设西南片区和高新创业城，力争已签约项目开工建设。加快重力石化装备项目建设，抓好国信科技产业园首期项目建设。加快粤西危化品应急救援基地、精细化工中试基地和石化产品运输车辆综合服务中心建设。推进水东港区建设原料码头工作。建设环保监测数据实时公布设施。

积极推进县域工业园区建设。出台《茂名市省级园区扩能增效专项资金股权投资方案》。加快园区道路、污水处理等基础设施建设。支持化州、茂南、滨海新区等地申报省级产业转移园。强化市对县域工业园区考核。

（三）乘势而上抓好交通基础设施建设。

乘势用劲抓好外通项目建设。包茂高速茂名段年底建成通车。全面开工建设汕湛高速茂名段。加快深茂铁路茂名段建设。加快推进云茂高速、茂名综合客货运枢纽、博贺疏港铁路和湛江机场迁建前期工作。加快推进河茂铁路电气化改造工程。

持续发力加快内联项目建设。加快建成茂化快线、茂东快线、高新大道、市民大道（二期）、悦民路和海洋大道东段工程。全面完成“迎国检”21项国省道路面改造工程。完成茂名港大道改线和延长线、博贺湾大道、恒大路改建工程。提升道路治超、养护、保洁、绿化和恢复排水系统等交通综合治理能力。实施公交优先战略，优化调整公交线路。

攻坚克难加快深水大港建设。加快推进粤电煤炭码头和博贺新港区东西防波堤工程。力争年内开工建设博贺新港区通用码头工程，抓紧推进中化液体化工码头项目和保利利丰码头项目前期工作。加快口岸通关服务中心和港口调度中心建设。

（四）着力推进中心城区扩容提质。

加强北组团现主城区城市建设和管理。加快完善东北五小区路网，修通西粤北路、官渡五六路，建设文明北路（官渡路至大园路段）、大园三路。启动站南片区开发。加快推进人民南五六路、粤秀北路、福地小区街坊路工程建设。完成官山一至四路、迎宾二路“黑底化”改造。完成高山桥改造工程。搬迁市政沥青搅拌站和水泥构件预制场。开展市区公园湖水治理工程，启动官渡公园建设，加快露天矿生态修复和公园建设。推动河西片区批发市场改造。开展精细化管理示范小区创建活动。研究出台城区临时泊车管理实施方案。落实门前“三包”责任制。加强户外广告经营管理。促进房地产市场健康发展。

加快南组团水东湾新城建设。继续抓好电白城区“七路一桥”建设，完成人民路、厂前路、新湖路和东、西湖改造工程。动工建设水东大道镇东段，推进新城大道建设，加快完成海滨大道相关工程，抓好海滨跨海大桥施工。加快水东湾综合整治开发项目建设，启动水东湾大桥主桥桩基施工，大力推进水东湾清淤整治工程。加快推进高地智慧城项目，推动路网工程建设。启动水东湾海洋公园（一期）工程建设。动工建设市人民医院水东湾新城院区。加快南海片区存量建设用地盘整开发步伐。

推进东组团博贺湾新城建设。全面启动博贺湾东片区土地一级开发和路网建设。加快保利东海岸项目配套设施建设。启动滨海大道东段和博贺湾大桥建设，推动电城、博贺两镇一体化。积极推进东湾绿城安置区、东湾公租房、高州人民医院滨海分院等配套服务项目建设。

（五）切实加强“三农”工作。

加快现代农业发展步伐。继续加大农业四大经营主体培育和发展。积极引进示范农作物新品种，推广农业先进实用新技术。加快南药产业发展，推进中国化橘红产业园项目。积极培育发展农产品电子商务，鼓励社会资本创办特色农产品网店，力争年内在淘宝网“中国特色馆”设立“茂名馆”。发展壮大农业龙头企业，加快农产品供销物流体系建设。健全市县镇三级农产品质量安全监管体系，建立农产品标识和可追溯制度。全面开展农村土地登记确权颁证工作。积极引导农村土地承包经营权依法流转，促进农业适度规模经营。抓好家禽“集中屠宰、冷链配送、生鲜上市”工作。完成高标准农田建设任务。

推进社会主义新农村建设。继续实施农村公益事业“一事一议”财政奖补项目。开展名镇名村示范村创建工作，抓好信宜市镇隆镇八坊村省级新农村示范片建设。推进信宜市创建国家级生态保护与建设示范区前期工作。扎实做好被征地农民权益保护。加强外出务工子弟学校投入和管理。改善炼油厂、乙烯厂及重大邻避设施周边农村水电路等生产生活设施。

（六）稳步推进改革创新。

深入推进行政体制改革。进一步精简行政审批事项，建立健全后续监管制度体系。加快推进行政审批服务标准化建设，建成全市统一的网上公共资源交易平台。全面推进清理行政职权和编制权责清单工作。推进行政执法体制改革，突出抓好城市管理综合行政执法改革。深化事业单位法人治理结构试点改革。落实工商、质监行政管理体制改革任务。

完善国资监管支持非公经济发展。构建国资监管“全覆盖”格局，将机关事业单位经营性国有资产和国有农贸市场纳入监管范围。加快完善公司法人治理结构。探索实施混合所有制改革。加快推进国有资产经营业绩考核工作。加大国有资产整合重组力度，引导支持主业突出、实力较强的国有企业兼并业务相关国有企业。坚持权利平等、机会平等、规则平等，消除各种隐性壁垒，支持非公有制经济健康发展。

积极推进财政体制改革。压减市直党政机关和参公管理事业单位日常公用经费和专项业务经费。适当集中县级新增财力，统筹用于市对各区（市）民生配套支出。加强非税收入统筹分配和规范管理，清理盘活财政存量资金。推广政府和社会资本合作（PPP）模式。稳步推进机关事业单位工作人员养老保险制度改革。年内完成全市党政机关公务用车制度改革。

强化金融改革创新。进一步推进农村金融改革创新综合试验区建设。力争2015年新增1家县域村镇银行。支持农信联社向农村商业银行转制改革。加快直接融资，推进中小企业上市。鼓励支持企业参与珠三角金融要素市场交易。探索开办具有茂名特色的农业保险新品种。落实“借用管还”的良性循环融资机制，防范债务风险。

（七）强化生态环境保护。

强化污染治理。做好污染源普查工作，制作污染源电子地图。深入开展高州水库生态保护试点工作，强化库区、东干渠、工业河周边污染源清理，保障饮用水安全。加快实施高州水库除险加固工程水库恢复89米正常蓄水淹没处理项目。深化小东江、白沙河流域污染整治，提高河流交界断面水质达标率。建成茂南污水处理厂、水东湾新城水质净化厂及14个中心镇污水处理设施。年内完成500家规模化畜禽养殖场治理任务。完成区（市）高污染燃料禁燃区划定工作，淘汰燃煤小锅炉。年内完成12960辆“黄标车”和老旧车辆淘汰任务。强化重点企业脱硫脱硝设施建设和监管。推动市区周边石油化工企业进入园区。开展工地扬尘、餐饮油烟、露天焚烧等大气污染整治。

加强节能降耗工作。全面完成“十二五”单位GDP能耗下降18%约束性指标。实施重点节能工程。抓好淘汰落后产能工作，严格实施固定资产投资项目节能评估审查制度，建立节能评估审查与能源消费总量控制共同约束机制。继续完善市级能源管理中心平台建设。

深入推进绿化茂名大行动。抓好公路绿化美化工作，完成省道370线白石至钱排路段、西城西路、茂化快线改线路段、茂东快线和茂名港大道南北段等道路绿化。抓好森林碳汇、生态景观林带、森林进城围城和乡村绿化美化四大工程，年内市辖城区改造若干街头小公园，打造钱排银杏景观林带。切实做好生态红线划定工作。

（八）切实保障和改善民生。

努力办好民生实事。抓好充分就业工作，全年实现城镇新增就业6万人，城镇失业人员再就业1.7万人，促进创业人数2400人，新增转移就业劳动力7万人，转移劳动力技能培训2.5万人。巩固社保扩面征缴成果，稳定并提高缴费率。力争城镇职工养老保险参保人数达100万人。切实提高社保待遇，城乡居民医保财政补助标准从320元提高至380元，提高企业职工养老保险待遇和城乡居民养老保险待遇。全面落实生育津贴等各项待遇。抓好底线民生工作，提高低保户、五保户、残疾人、孤儿保障水平。加大保障房建设力度，努力解决住房困难家庭及特殊人群住房问题。加快“两不具备”村庄搬迁安置工作。全面完成全市2886户大中型水库移民特困户、五保户安居工程建设任务。力争年内建成市颐年苑养老服务综合楼，全市实现各类收养性养老床位2万张。进一步加快地质灾害隐患点搬迁治理工作。深入推进新一轮扶贫开发“双到”工作。继续抓好城乡清洁工程。

大力提升科教文卫体事业发展水平。实施创新驱动发展战略，扎实推进国家知识产权试点城市建设。加快教育创强和义务教育均衡县创建工作，实现教育强市目标和义务教育均衡县全覆盖。落实主城区义务教育学校布点，开工建设市愉园小学。实施残疾学生15年免费教育。创建广东茂名健康职业学院，筹建广东茂名幼儿师范高等专科学校。继续深化医药卫生体制改革，抓好各区（市）公立医院综合改革工作。整合卫生计生服务资源，完善镇级卫生院软硬件建设。加强人口计生工作。推进公共文化体育建设，打造文化活动品牌。加快乡镇农民健身工程建设，广泛开展全民健身活动。大力引进急需紧缺专业人才，注重培养实用型人才。

加强和创新社会治理。扎实推进“平安茂名”建设，推进治安重点地区集中整治，加大电信诈骗、涉黑涉毒违法犯罪和涉民生食品安全犯罪等专项治理力度。加大“六五”普法力度，深化“一村居一律师”工作。加强网络问政，建设清朗网络空间。加快社会组织培育发展。加强突发事件隐患排查和预警发布，提高应急管理能力和水平。加强安全生产基层基础建设。完善食品药品监管体制改革，建设市食品药品检验检测中心。加大打假治劣工作力度。做好国防动员教育、双拥、优抚安置工作，维修改造茂名市烈士纪念碑及其园区。

“十三五”是我市爬坡过坎的关键时期，做好“十三五”规划编制工作意义重大。要强化战略思维，高水平、高质量编制好“十三五”规划纲要以及重点专项规划。

各位代表！加强政府自身建设，是全面完成今年各项目标任务的重要保证。市政府将按照为民务实清廉的要求，振奋精神，扎实工作，努力建设法治政府、服务政府、高效政府和廉洁政府，提升政府公信力。一是加强民主法治建设。认真落实人大及其常委会各项会议决定，定期向人大及其常委会报告工作，自觉接受监督，主动加强和人民政协的联系并向其通报工作。认真办理人大代表建议和政协委员提案。广泛听取各民主党派、工商联、无党派人士和各人民团体意见。切实维护宪法和法律权威，提高依法行政水平。二是大力弘扬实干精神。强化责任意识，明确责任，细化目标，形成工作台账，使人人有压力。强化“钉钉子”精神，牢牢盯住重点工作和重点项目，深入现场一线，反复督促检查，实实在在把一件件事做好。强化担当精神，不回避问题、不推诿扯皮，敢于啃“硬骨头”，想方设法把事情办好。注重用结果说话，进一步完善利益导向机制，形成你追我赶、争先进位的良好工作氛围。三是加强廉政建设。深入落实中央八项规定、“三严三实”、国务院“约法三章”等要求，巩固拓展党的群众路线教育实践活动成果，保持整治“四风”高压态势。强化审计在政府治理中的重要保障作用。落实党风廉政建设责任制，深入推进政务公开，完善重点领域关键环节廉政风险防控，坚决查处腐败案件。

各位代表，展望新的一年，我们信心满怀！让我们在市委的坚强领导下，锐意进取、真抓实干、改革创新、奋发图强，加快推动茂名振兴发展，全力开创茂名更加美好的明天！
